# Differential Chromosome- and Plasmid-Borne Resistance of *Escherichia coli hfq* Mutants to High Concentrations of Various Antibiotics

**DOI:** 10.3390/ijms22168886

**Published:** 2021-08-18

**Authors:** Lidia Gaffke, Krzysztof Kubiak, Zuzanna Cyske, Grzegorz Węgrzyn

**Affiliations:** Department of Molecular Biology, University of Gdansk, Wita Stwosza 59, 80-308 Gdansk, Poland; lidia.gaffke@ug.edu.pl (L.G.); krzypl@gmail.com (K.K.); zuzanna.cyske@phdstud.ug.edu.pl (Z.C.)

**Keywords:** antibiotic resistance, *Escherichia coli hfq* gene, ColE1-like plasmids

## Abstract

The Hfq protein is a bacterial RNA chaperone, involved in many molecular interactions, including control of actions of various small RNA regulatory molecules. We found that the presence of Hfq was required for survival of plasmid-containing *Escherichia coli* cells against high concentrations of chloramphenicol (plasmid p27cmr), tetracycline (pSC101, pBR322) and ampicillin (pBR322), as *hfq*^+^ strains were more resistant to these antibiotics than the *hfq*-null mutant. In striking contrast, production of Hfq resulted in low resistance to high concentrations of kanamycin when the antibiotic-resistance marker was chromosome-borne, with deletion of *hfq* resulting in increasing bacterial survival. These results were observed both in solid and liquid medium, suggesting that antibiotic resistance is an intrinsic feature of these strains rather than a consequence of adaptation. Despite its major role as RNA chaperone, which also affects mRNA stability, Hfq was not found to significantly affect *kan* and *tet* mRNAs turnover. Nevertheless, *kan* mRNA steady-state levels were higher in the *hfq*-null mutant compared to the *hfq*^+^ strain, suggesting that Hfq can act as a repressor of *kan* expression.This observation does correlate with the enhanced resistance to high levels of kanamycin observed in the *hfq*-null mutant. Furthermore, dependency on Hfq for resistance to high doses of tetracycline was found to depend on plasmid copy number, which was only observed when the resistance marker was expressed from a low copy plasmid (pSC101) but not from a medium copy plasmid (pBR322). This suggests that Hfq may influence survival against high doses of antibiotics through mechanisms that remain to be determined. Studies with pBR322Δ*rom* may also suggest an interplay between Hfq and Rom in the regulation of ColE1-like plasmid replication. Results of experiments with a mutant devoid of the part of the *hfq* gene coding for the *C*-terminal region of Hfq suggested that this region, as well as the *N*-terminal region, may be involved in the regulation of expression of antibiotic resistance in *E. coli* independently.

## 1. Introduction

The Hfq protein has been discovered in studies on *Escherichia coli* bacteriophage Qβ as a factor required for RNA replication of this virus [1]. Subsequent studies indicated that this small protein, encoded by the *hfq* gene, interacts with various RNA species, acting as an RNA chaperone. This activity results in regulation of expression of many genes, especially at the stage of post-transcriptional modulations of RNA-RNA interactions and controlling availability of mRNA molecules to the translation process (reviewed in [2,3,4]).

**Figure 1:**
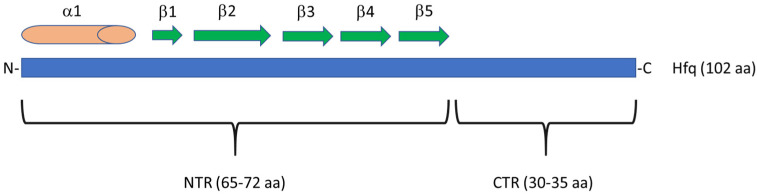
Scheme of the Hfq protein of *E. coli*. This protein consists of 102 aa residues that can be divided into two regions, NTR and CTR. In NTR, one α helix and five β strands can be distinguished. See [5] for more details. The scheme is not drawn to scale.

Apart from its RNA chaperone activities, previously unknown properties of Hfq were identified recently. The *E. coli* Hfq protein is composed of 102 amin acid (aa) residues and consists of two structural regions, called the *N*-terminal region (NTR) and the *C*-terminal region (CTR) (Figure 1). NTR (about 65 aa residues, though sometimes it is suggested to cover 72 aa) contains the capping α-helical structure and five β-stands, and it is responsible for interactions with RNA molecules (reviewed in [2,3,4,5]). CTR is a relatively small region (30–35 aa residues) that is less conserved in evolution than NTR and reveals interesting properties both related and unrelated to RNA binding [5]. The RNA-related activity of CTR has been proposed to be required for modulating interactions between mRNA and small RNA molecules [6]. This was demonstrated for CTR originating from *E. coli* and *Caulobacter crescentus* [7]. 

Although the role of CTR was puzzling for many years, recent studies indicated that it may play important biological functions not only in RNA binding [6,7], but also in DNA transactions. First, it was demonstrated that CTR is able to assemble into an amyloid-like structure [8]. Then, this region of Hfq was shown to be involved in DNA binding, resulting in compaction and condensation of DNA [9]. Mutations in the *hfq* gene resulted in changes in plasmid DNA supercoiling in *E. coli* [10], suggesting that this might be partially responsible for negative effects of *hfq* dysfunction on ColE1-like plasmid DNA replication, as observed previously [11]. Moreover, CTR causes a local alignment of nucleoprotein fibers, consisting of Hfq and DNA, which affects the double helix structure [12]. Very recently, Hfq was found to be able to bind to G-quadruplex and to stabilize this structure [13]. This Hfq activity may affect mechanical properties of the bacterial chromosome [14], which can be involved in modulation of genomic instability [15].

Since Hfq is involved in the regulation of expression of many genes, it is not a surprise that various properties of bacteria related to their virulence and antibiotic resistance can be modulated by this protein. In fact, the Hfq protein participates in the development of virulence of various pathogens, including *Yersinia pestis, Salmonella, Bacillus anthracis, Actinobacillus pleuropneumoniae, Bordetella pertussis, Pseudomonas aeruginosa, Listeria monocytogenes,* and others, through influencing gene expression regulation [16,17,18,19,20,21,22,23]. Regarding antibiotic susceptibility, in *E. coli*, Hfq regulates expression (at post-transcriptional level) of genes coding for proteins of the efflux system, stimulating multidrug resistance [24]. Such regulations include Hfq-mediated control of actions of small RNAs, which participate in bacterial response to antibiotic treatment [25]. This issue has been reviewed and discussed in detail [26]. Moreover, in *Aeromonas veronii*, the Hfq protein mediates persistence to various antibiotics [27]. In *P. aeruginosa*, deletion of the *hfq* gene significantly increased bacterial susceptibility to different classes of antibiotics due to changes in regulation of expression of various genes involved in development of resistance to these antibacterial agents [28]. When sublethal concentrations of neomycin, paromomycin or kanamycin were used, *E. coli* growth was impaired more efficiently in *hfq* mutant relative to wild-type cells. Since Hfq was demonstrated to be involved in ribosome biogenesis, due to interactions with the 17S rRNA and participation in maturation of 16S rRNA, it was suggested that increased susceptibility of *hfq* mutants to aminoglycoside antibiotics may be related to this involvement of Hfq in this process [29].

Despite published works that indicate effects of Hfq on multiple antibiotic resistance in bacteria, as summarized above, to our knowledge, no reports are available on possible implications of the *hfq* gene function in plasmid-borne antibiotic resistance. Expression of genes located on multicopy plasmids may occur at levels significantly higher than that of chromosomal genes, thus, we hypothesize that resistance to high concentrations of antibiotics might be affected by *hfq* function, especially since Hfq has been implicated in the control of replication of ColE1-like plasmid DNA replication [11]. Moreover, Hfq was proposed recently as a novel target for antimicrobial drugs [30]. Therefore, in this work, we tested the effects of mutations in the *hfq* gene on chromosome-borne and plasmid-borne resistance of *E. coli* to high concentrations of different antibiotics. 

## 2. Results

**Figure 2:**
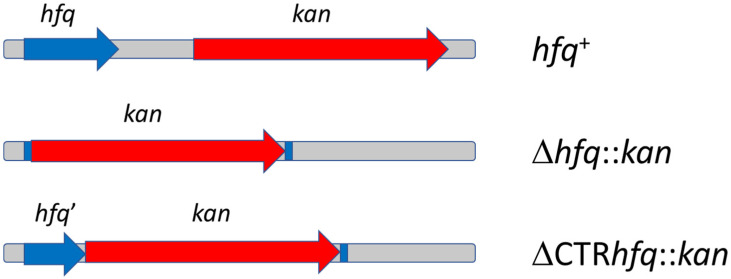
Schematic representation of genomic regions of *E. coli* strains *hfq*^+^, Δ*hfq*::*kan*, and ΔCTR*hfq*::*kan*. Whole length *hfq* gene, and its truncated form, *hfq’*, coding for intact NTR, are shown as blue arrows. The *kan* gene, responsible for kanamycin resistance, is marked by red arrow. The scheme is not drawn to scale.

In this study, three otherwise isogenic *E. coli* strains, derivatives of MG1655, were used. They bear either wild-type *hfq* allele (*hfq*^+^) and the *kan* gene located near the *hfq* locus, Δ*hfq*::*kan* mutation (the *hfq* gene replaced with *kan*), and ΔCTR*hfq*::*kan* (intact *hfq* fragment coding for the NTR72 domain, and *hfq* fragment coding for CTR replaced with *kan*). The *hfq* regions of genomes of these strains are presented schematically in Figure 2. These strains were either plasmid-free or were transformed with p27cmr, pSC101, pBR322 or pBR332Δ*rom* plasmids (see Table 1 and Section 4.2 for features of these plasmids). 

We assessed bacterial resistance to antibiotics at their concentrations significantly higher relative to standard concentrations used in laboratory practice. Concentrations of particular antibiotics were chosen as those causing about 50% decrease in survival by at least one of the tested strains. Bacteria were cultured without an antibiotic and then plated on Petri dishes with solid medium containing either no antibiotic or its various concentrations. 

### 2.1. Chromosome-Borne Resistance to Kanamycin

Under the experimental conditions described above, we found that chromosome-borne kanamycin resistance was drastically decreased in the *hfq*^+^ strain, relative to Δ*hfq*::*kan* and ΔCTR*hfq*::*kan* mutants, with Δ*hfq*::*kan* bacteria revealing the highest resistance (Table 2). These results indicate that the *hfq* function interferes with expression of kanamycin resistance at the high (233 μg/mL) antibiotic concentration.

We also investigated the growth of *hfq*^+^, Δ*hfq*::*kan*, and ΔCTR*hfq*::*kan* strains in liquid cultures to which a high concentration of kanamycin (233 μg/mL) was added. Particularly, we asked if the transfer of bacteria, revealing resistance to high antibiotic concentration on a solid medium from a liquid broth devoid of antibiotic, to that containing its high concentration results in high mortality and then recovery of the cell population, or if such resistance is an intrinsic feature of the bacterial strain and the cell population is already resistant and not affected? This may be important in the light of recent discussion on distinguishing between resistance, tolerance, and persistence to antibiotic treatment of bacteria [31].

**Figure 3:**
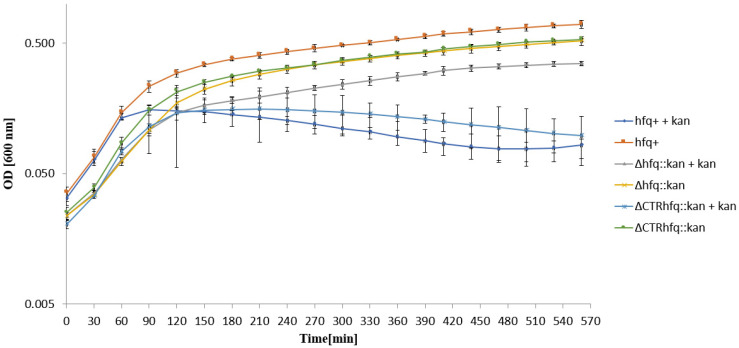
Growth of *E. coli* strains *hfq*^+^, Δ*hfq*::*kan*, and ΔCTR*hfq*::*kan* in the LB liquid medium containing 233 μg/mL kanamycin (+kan) or devoid of this antibiotic (no mark) at 37 °C, after transferring bacteria from antibiotic-free LB and diluting the culture 10 times in a fresh LB medium (with or without kanamycin) at time 0. The presented data indicate mean values from three independent experiments with error bars showing SD. Minimal inhibitory concentrations (MIC) of kanamycin for *hfq*^+^, Δ*hfq*::*kan*, and ΔCTR*hfq*::*kan* strains under these conditions were 64, 512, and 256 μg/mL, respectively.

When *hfq*^+^ strain (bearing the *kan* gene near the wild-type *hfq* allele) was transferred from antibiotic-free liquid LB medium to that containing 233 μg/mL kanamycin, its growth was halted within 90 min (Figure 3), as could be expected on the basis of results of experiments with solid media (presented in Table 2). Similar behavior was observed for the ΔCTR*hfq*::*kan* mutant, which also revealed poor survival on a solid medium with the high antibiotic level. However, Δ*hfq*::*kan* continued to grow in the liquid medium with 233 μg/mL kanamycin immediately after their transfer from an antibiotic-free medium (Figure 3). This indicates that no adaptation period was required and that resistance to high kanamycin concentration is an intrinsic feature of this Δ*hfq* mutant bearing the *kan* gene.

### 2.2. Transformation Efficiency of hfq^+^ and hfq Mutant Hosts with Plasmids

In the next step, we investigated plasmid-borne antibiotic resistance of *E. coli* cells bearing different *hfq* alleles. Bacterial strains were transformed with plasmids p27cmr (bearing a chloramphenicol resistance gene, *cat*), pSC101 (bearing a tetracycline resistance gene, *tet*), pBR322 (a ColE-like replicon, bearing tetracycline and ampicillin resistance genes, *tet* and *bla*), and a derivative of pBR322 with deletion of the *rom* gene, coding for a negative regulator of plasmid DNA replication (pBR332Δ*rom* plasmid, used as a control in the transformation efficiency assay) (see Table 1 for details). We assessed the efficiency of transformation of bacterial strains with plasmids, which is commonly considered as a rough measure of ability of a plasmid to replicate effectively in the host strain. Results of these experiments are presented in Table 3.

All plasmids successfully transformed all recipients strains, however, efficiency of transformation was lower for *hfq* mutants. These results corroborated previously published suggestions that replication of some plasmids, particularly those belonging to the ColE1 family, can be affected by Hfq dysfunction [9]. The *rom* gene of ColE1-like plasmids, like pBR322, codes for a negative regulator of replication initiation, thus, increased efficiency of transformation with this plasmid, relative to wild-type counterpart, supports the assumption that efficiency of transformation can be used as a rough estimation of plasmid replication effectiveness. Interestingly, in the absence of the *rom* function in pBR322, there were considerable differences between efficiency of transformation of Δ*hfq*::*kan* and ΔCTR*hfq*::*kan* mutants, contrary to the wild-type ColE1-like replicon (Table 3). This suggests a possible interplay between Hfq and Rom in the regulation of pBR322 plasmid replication regulation.

### 2.3. Low Copy Number Plasmid p27cmr (Lambdoid)-Borne Resistance to Chloramphenicol

We tested p27cmr-borne resistance to a high dose (204 μg/mL) of chloramphenicol of *hfq*^+^, Δ*hfq*::*kan,* and ΔCTR*hfq*::*kan* hosts. It was found that the resistance of both *hfq* mutants was significantly impaired relative to the wild-type strain, while the effect of the decreased resistance was more pronounced in the Δ*hfq*::*kan* bacteria (Table 4). These results indicate that the *hfq* gene plays a considerable role in the expression of plasmid-borne resistance to high levels of chloramphenicol.

**Figure 4:**
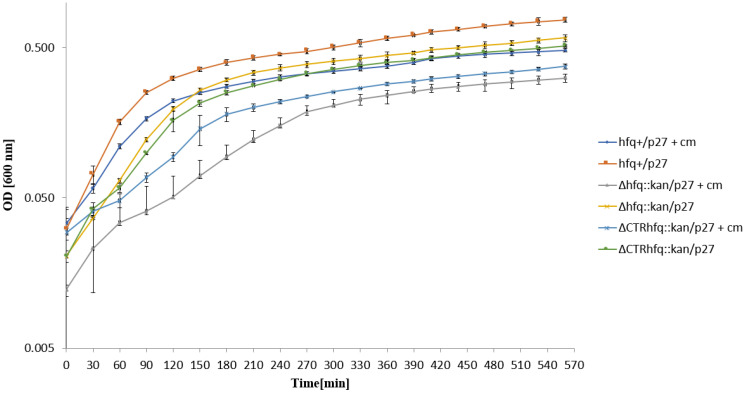
Growth of *E. coli* strains *hfq*^+^, Δ*hfq*::*kan*, and ΔCTR*hfq*::*kan* bearing plasmid p27cmr (marked as p27) in the LB liquid medium containing 204 μg/mL chloramphenicol (+cm) or devoid of this antibiotic (no mark) at 37 °C, after transferring bacteria from antibiotic-free LB and diluting the culture 10 times in a fresh LB medium (with or without chloramphenicol) at time 0. The presented data indicate mean values from three independent experiments with error bars showing SD. Minimal inhibitory concentrations (MIC) of chloramphenicol for *hfq*^+^, Δ*hfq*::*kan*, and ΔCTR*hfq*::*kan* hosts bearing p27cmr under these conditions were >2048, 1024, and 1024 μg/mL, respectively.

When testing growth in the liquid LB medium with a high dose (204 μg/mL) of chloramphenicol after a transfer of bacteria from antibiotic-free broth, we found that *hfq*^+^, Δ*hfq*::*kan,* and ΔCTR*hfq*::*kan* hosts bearing p27cmr continued to grow immediately, though growth rates of *hfq* mutants were considerably lower at initial stages of the experiment (Figure 4), most probably due to lower fractions of survivors, as observed on solid medium (Table 4). These results indicated that, similarly to chromosome-born kanamycin resistance, the resistance to high chloramphenicol concentration (204 μg/mL), caused by expression of the *cat* gene located on the p27cmr plasmid, is an intrinsic feature of bacteria rather than a kind of developing antibiotic tolerance.

### 2.4. Low Copy Number Plasmid pSC101-Borne Resistance to Tetracycline

When comparing pSC101-borne resistance to high concentrations of tetracycline, we found an inability of the Δ*hfq*::*kan*/pSC101 strain to form colonies in the presence of 68 μg/mL of this antibiotic, while both *hfq*^+^ and ΔCTR*hfq*::*kan* bacteria bearing pSC101 (a low copy number plasmid) could grow effectively under these conditions (Table 5). Therefore, it appears that NTR of Hfq may play a crucial role in development of pSC101-borne resistance to a high (68 μg/mL) concentration of tetracycline. 

**Figure 5:**
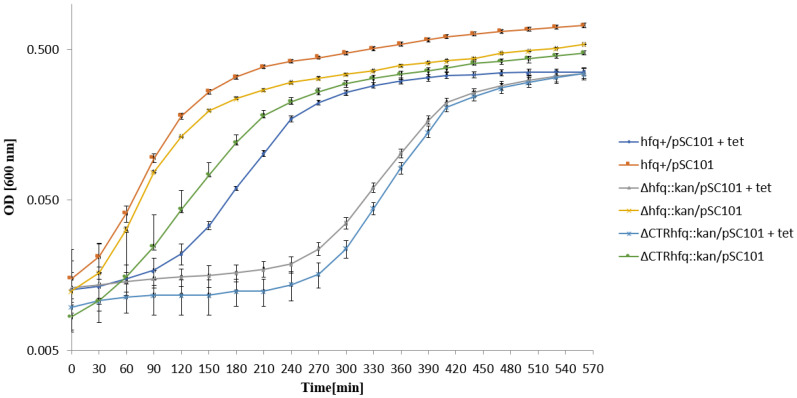
Growth of *E. coli* strains *hfq*^+^, Δ*hfq*::*kan*, and ΔCTR*hfq*::*kan* bearing plasmid pSC101 in the LB liquid medium containing 68 μg/mL tetracycline (+tet) or devoid of this antibiotic (no mark) at 37 °C, after transferring bacteria from antibiotic-free LB and diluting the culture 10-times in a fresh LB medium (with or without tetracycline) at time 0. The presented data indicate mean values from three independent experiments with error bars showing SD. Minimal inhibitory concentrations (MIC) of tetracycline for *hfq*^+^, Δ*hfq*::*kan*, and ΔCTR*hfq*::*kan* hosts bearing pSC101 under these conditions were >2048, 1024, and >2048 μg/mL, respectively.

Interestingly, different behaviors were observed for *hfq*^+^/pSC101 and ΔCTR*hfq*::*kan*/pSC101, both revealing similar phenotypes on solid medium, after transferring to LB liquid medium with 68 μg/mL tetracycline (Figure 5). The *hfq*^+^/pSC101 started to grow immediately after the transfer, while growth of the ΔCTR*hfq*::*kan*/pSC101 mutant was initially inhibited, and restored only after 5 h incubation. We concluded that resistance of *hfq*^+^/pSC101 to 68 μg/mL tetracycline is an intrinsic feature of this strain, whereas otherwise isogenic ΔCTR*hfq*::*kan*/pSC101 mutant required a long adaptation time. Intriguingly, after a long (5 h) initial inhibition of growth of the Δ*hfq*::*kan*/pSC101 mutant, which was not able to form colonies on solid medium containing 68 μg/mL tetracycline, efficient bacterial growth was observed, suggesting either isolation of a suppressor mutant or recovery of growth that was possible in liquid broth but not on a solid medium (Figure 5).

### 2.5. Medium and High Copy Number ColE1-like Plasmid-Borne Resistance to Tetracycline

Different patterns of resistance to high concentration (68 μg/mL) of tetracycline were found when all tested host strains were transformed with a medium copy number plasmid pBR322. In this experiment, the resistance was comparable for *hfq*^+^, Δ*hfq*::*kan*, and ΔCTR*hfq*::*kan* strains (Table 6).

**Figure 6:**
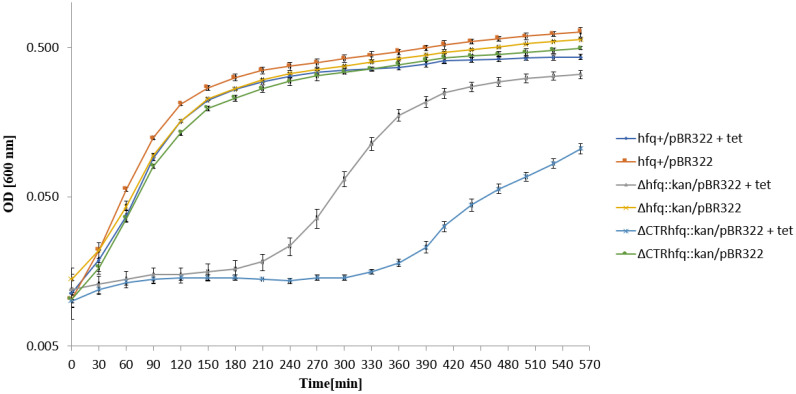
Growth of *E. coli* strains *hfq*^+^, Δ*hfq*::*kan*, and ΔCTR*hfq*::*kan* bearing plasmid pBR322 in the LB liquid medium containing 68 μg/mL tetracycline (+tet) or devoid of this antibiotic (no mark) at 37 °C, after transferring bacteria from antibiotic-free LB and diluting the culture 10 times in a fresh LB medium (with or without tetracycline) at time 0. The presented data indicate mean values from three independent experiments with error bars showing SD. Minimal inhibitory concentrations (MIC) of tetracycline for *hfq*^+^, Δ*hfq*::*kan*, and ΔCTR*hfq*::*kan* hosts bearing pBR322 under these conditions were 512, 256, and 1024 μg/mL, respectively.

While growth of the *hfq*^+^/pBR322 strain in the liquid LB medium containing 68 μg/mL tetracycline started immediately after the transfer from an antibiotic-devoid medium, otherwise isogenic *hfq* mutants, Δ*hfq*::*kan* and ΔCTR*hfq*::*kan* bearing pBR322, required a relatively long time to restore the growth under these conditions, with ΔCTR*hfq*::*kan*/pBR322 being more efficiently inhibited (Figure 6). Since this phenomenon was similar to results presented for strains bearing the pSC101 plasmid under the high tetracycline level pressure, one might suggest that resistance to this antibiotic is expressed in a specific, somewhat impaired, way in *hfq* mutants.

**Figure 7:**
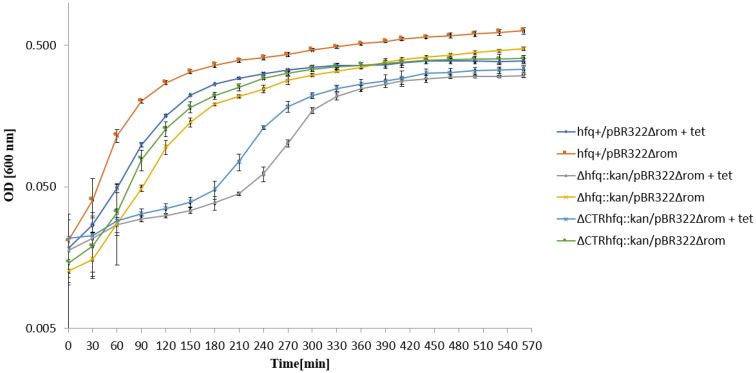
Growth of *E. coli* strains *hfq*^+^, Δ*hfq*::*kan*, and ΔCTR*hfq*::*kan* bearing plasmid pBR322Δ*rom* in the LB liquid medium containing 68 μg/mL tetracycline (+tet) or devoid of this antibiotic (no mark) at 37 °C, after transferring bacteria from antibiotic-free LB and diluting the culture 10 times in a fresh LB medium (with or without tetracycline) at time 0. The presented data indicate mean values from three independent experiments with error bars showing SD. Minimal inhibitory concentrations (MIC) of tetracycline for *hfq*^+^, Δ*hfq*::*kan*, and ΔCTR*hfq*::*kan* hosts bearing pBR322Δ*rom* under these conditions were 256, 128, and 128 μg/mL, respectively.

To assess whether the delay in growth restoration of *hfq* mutants bearing pBR322 after the transfer to the liquid medium containing 68 μg/mL tetracycline depends on efficiency of plasmid replication, we have repeated the experiment using a plasmid variant devoid of the *rom* gene (pBR322Δ*rom*), coding for a negative regulation of the plasmid replication initiation. No influence of the Δ*rom* mutation on the growth of the *hfq*^+^ host could be observed under these conditions (Figure 7). A delay was still observed for Δ*hfq*::*kan* and ΔCTR*hfq*::*kan* hosts, though the effect was less pronounced relative to these mutants bearing pBR322 (Figure 7). These results indicate that in principle, the delay in bacterial growth depends on the kind of antibiotic, however, combination of the absence of the Rom protein and Δ*hfq*::*kan* or ΔCTR*hfq*::*kan* mutantions weakens this phenomenon to some extent. This may suggest an interplay between Hfq and Rom in the regulation of ColE1-like plasmid DNA replication, corroborating the hypothesis on the influence of the *hfq* gene function on this process through functional interactions with Rom.

### 2.6. Medium and High Copy Number ColE1-like Plasmid-Borne Resistance to Ampicillin

Using the same set of strains, bearing pBR322, we assessed the resistance of high concentration (5 mg/mL) of ampicillin. In these experiments, resistance of the Δ*hfq*::*kan* strain was significantly (though not drastically) lower than those observed in *hfq*^+^ and ΔCTR*hfq*::*kan* strains, while results obtained for the two latter strains were similar (Table 7). Again, these results suggest involvement of CTR of Hfq in development of resistance to high antibiotic concentrations.

**Figure 8:**
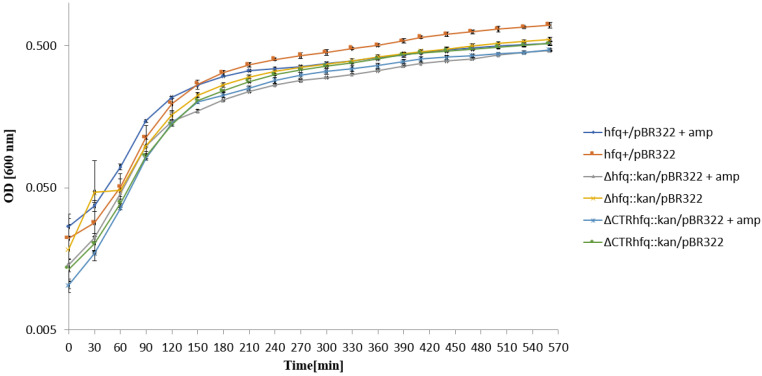
Growth of *E. coli* strains *hfq*^+^, Δ*hfq*::*kan*, and ΔCTR*hfq*::*kan* bearing plasmid pBR322 in the LB liquid medium containing 5 mg/mL ampicillin (+amp) or devoid of this antibiotic (no mark) at 37 °C, after transferring bacteria from antibiotic-free LB and diluting the culture 10 times in a fresh LB medium (with or without ampicillin) at time 0. The presented data indicate mean values from three independent experiments with error bars showing SD. Minimal inhibitory concentration (MIC) of ampicillin for *hfq*^+^, Δ*hfq*::*kan*, and ΔCTR*hfq*::*kan* hosts bearing pBR322 under these conditions was >2048 μg/mL for each strain.

**Figure 9:**
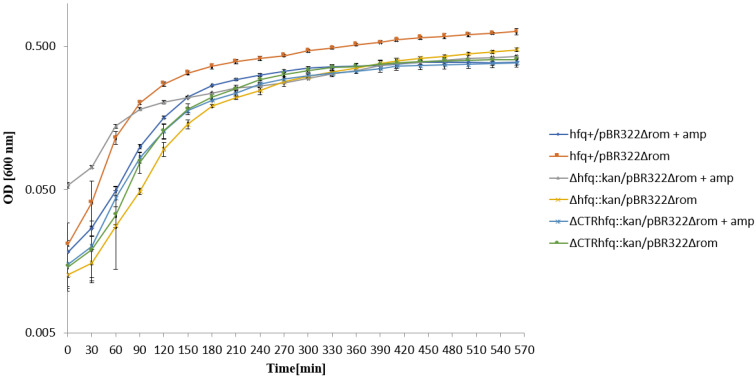
Growth of *E. coli* strains *hfq*^+^, Δ*hfq*::*kan*, and ΔCTR*hfq*::*kan* bearing plasmid pBR322Δ*rom* in the LB liquid medium containing 5 mg/mL ampicillin (+amp) or devoid of this antibiotic (no mark) at 37 °C, after transferring bacteria from antibiotic-free LB and diluting the culture 10 times in a fresh LB medium (with or without ampicillin) at time 0. The presented data indicate mean values from three independent experiments with error bars showing SD. Minimal inhibitory concentration (MIC) of ampicillin for *hfq*^+^, Δ*hfq*::*kan*, and ΔCTR*hfq*::*kan* hosts bearing pBR322 Δ*rom* under these conditions was >2048 μg/mL each strain.

In the liquid LB medium with 5 mg/mL ampicillin, all tested strain bearing pBR322 started to grow immediately after the transfer from the medium with no antibiotic (Figure 8). These results confirmed that the growth characteristics of *hfq* mutants under such conditions depend on the kind of antibiotic used at high concentration. As could be expected, deletion of the *rom* gene from the plasmid did not influence bacterial growth in the liquid medium with 5 mg/mL ampicillin (Figure 9).

### 2.7. Copy Number of Plasmid pBR322 in hfq^+^ and hfq Mutant Hosts at Various Growth Phases

To test whether pBR322 plasmid copy number varies between all tested strains, quantitative real time PCR technique was employed. Since previous studies indicated that levels of the Hfq protein are higher at the stationary phase of bacterial growth [9], bacteria were cultured in a liquid medium and samples were withdrawn at exponential phase of growth (OD_600_ = 0.3), at the late exponential/entrance to the stationary phase (OD_600_ = 0.8) and at the stationary phase (OD_600_ = 1.4).

When assessing pBR322 plasmid copy number, we found that it was the highest at the late exponential/early stationary phase of growth (Table 8). However, these values were not related to the function of the *hfq* gene, as they were comparable in all tested *E. coli* host strains (Table 8).

### 2.8. Decay of mRNAs Derived from Tet and Kan Genes in hfq^+^ and hfq Mutant Hosts

Since Hfq is known to interact with RNA molecules [2,3,4], we asked whether the stability of selected mRNAs encoding antibiotic-resistance proteins is affected in *hfq* mutants. We chose *tet* and *kan* genes as effects of the Δ*hfq*::*kan* mutation on resistance to high concentrations of tetracycline and kanamycin were opposite relative to *hfq*^+^ bacteria (drastically decreased pSC101-borne resistance to tetracycline and chromosome-born high resistance to kanamycin of the Δ*hfq*::*kan* mutant; Table 5 and Table 2, respectively). Steady-state levels of *tet* and *kan* mRNAs and stability of these molecules (after rifampicin-mediated inhibition of transcription) were determined using the Northern-blotting method.

**Figure 10:**
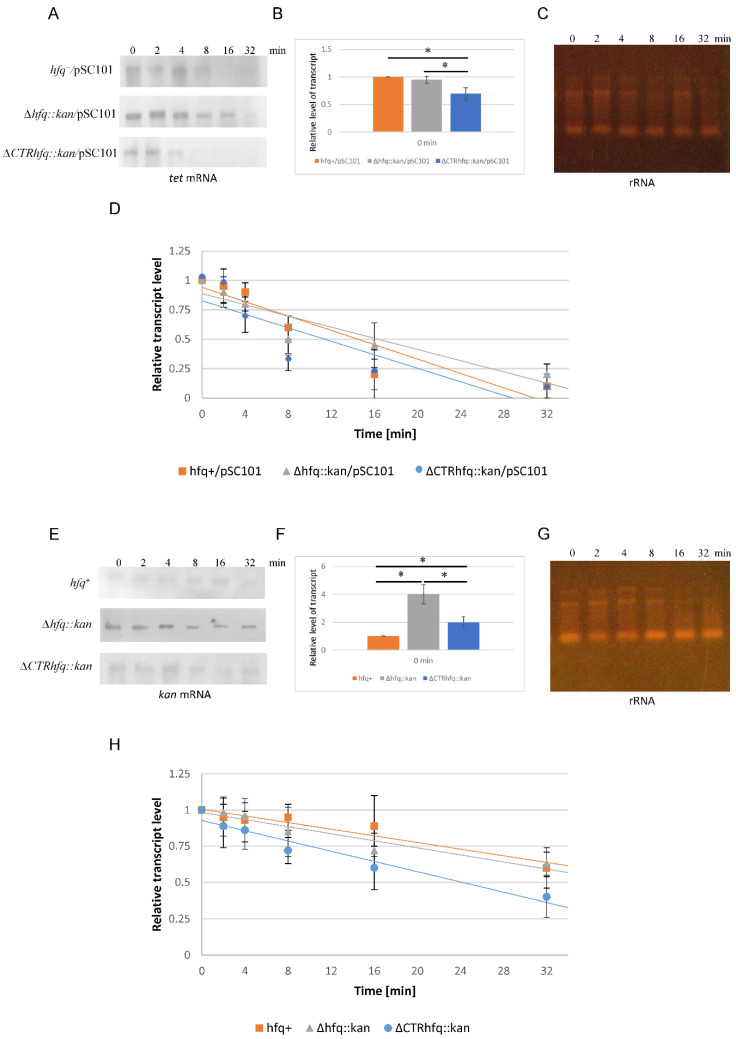
Levels and stability of *tet* (**A**–**D**) and *kan* (**E**–**H**) mRNAs in *E. coli* strains *hfq*^+^, Δ*hfq*::*kan*, and ΔCTR*hfq*::*kan* bearing either pSC101 (**A**–**D**) or no plasmid (**E**–**H**), as estimated by Northern-blotting. Representative blots (**A**,**E**) and quantification of results (**B**,**D**,**F**,**H**) are presented. Levels of mRNAs were calculated relative to internal controls—rRNA levels (representative of ethidium bromide-stained gels) are shown in panels (**C**,**G**). Values presented in panels (**B**,**D**,**F**,**H**) are mean values from three independent experiments with error bars showing SD. Statistically significant differences (*p* < 0.05) between results presented in panels (**B**,**F**) are marked by asterisks.

Levels of pSC101-derived *tet* mRNA under steady-state conditions were similar in *hfq*^+^ and Δ*hfq*::*kan* bacteria, while decreased in the ΔCTR*hfq*::*kan* mutant (Figure 10B). This was irrespective of the increased stability of the mRNA in the Δ*hfq*::*kan* strain (half-life of 16 min) and its decreased stability in the ΔCTR*hfq*::*kan* strain (half-life of 11 min), relative to *hfq*^+^ (half-life of 14.5 min) (Figure 10). No simple correlation between resistance of pSC101-harboring bacteria to high concentration (68 μg/mL) of tetracycline and *tet* mRNA level or stability could be found. Thus, the mechanism of this phenomenon remains to be elucidated, but one might speculate about putative involvement of Hfq in regulating expression of the *tet* gene at one of the levels different to transcription and/or RNA stability.

The level of *kan* mRNA under steady-state conditions was higher in both *hfq* mutants than in the *hfq*^+^ strain, however, it was about two times higher in Δ*hfq*::*kan* than in ΔCTR*hfq*::*kan* bacteria (Figure 10F). When estimating the half-life of this mRNA, similar values (35 min) could be estimated for *hfq*^+^ and Δ*hfq*::*kan* strains, while this transcript was degraded more rapidly (half-life of 24 min) in the ΔCTR*hfq*::*kan* mutant (Figure 10E,G,H). These results indicated that the resistance of bacteria (bearing the *kan* gene in the chromosome) to high concentration (233 μg/mL) of kanamycin (Table 1) correlates directly with steady-state levels of *kan* mRNA (Figure 10F) rather than with its stability. This suggests that Hfq may influence the expression of the *kan* gene not only by regulating mRNA degradation, but also by yet unknown mechanism(s).

### 2.9. Levels of Hfq and NTRHfq Proetins in hfq^+^ and ΔCTRhfq::kan Strains, Respectively

To test the levels of the whole-length Hfq protein and its NTR, we performed Western-blotting experiments. As expected, Hfq could not be detected in the Δ*hfq*::*kan* strain, while levels of the whole-length Hfq (in the *hfq*^+^ strain) and NTR of this protein (in the ΔCTR*hfq*::*kan* mutant) were not significantly different (data not shown). These results indicated, as expected, that the differences observed between investigated strains did not arise from different expression of various *hfq* alleles.

## 3. Discussion

Although the Hfq protein has been indicated as a factor involved in development of bacterial virulence and modulation of microbial response to antibiotics [16,17,18,19,20,21,22,23,24,25,26,27], and this protein has been proposed as a novel target for antibacterial drugs [30], the question about its functions in expressing resistance to high concentrations of antibiotics has not been addressed previously. On the other hand, one can imagine that high concentrations of antibiotics may occur locally in the natural environment (where they are produced by some microorganisms to compete with others) and in human or animal gastrointestinal tract shortly after oral administration of antimicrobial drugs. In addition, one can also imagine that the presence of antibiotic resistance genes on plasmids may cause their high level of expression and development of resistance to antibiotic concentrations considerably higher than that found in the case of chromosome-borne resistance. Knowing that Hfq is involved in regulation of expression of many genes in bacterial cells, including those involved in response to antibiotics [24,25,26,27], and that it can be involved in the control of plasmid DNA replication [11], determination of chromosome- and plasmid-borne resistance to antibiotics appeared important and reasonable.

Since both *hfq*^+^ and *hfq* mutant strains revealed similar resistance to various antibiotics at their standard concentrations, irrespective of if an antibiotic resistance gene was located in the chromosome or in a plasmid (Table 2, Table 4, Table 5, Table 6 and Table 7), we tested the effects of significantly increased concentrations of these compounds. The tested concentrations were chosen as those causing about 50% decrease in survival of at least one of the tested strains, in our preliminary experiments.

Perhaps surprisingly, we found a drastically decreased chromosome-borne resistance to high (233 μg/mL) kanamycin concentration in the *hfq*^+^ strain relative to *hfq* mutants (Table 2). Interestingly, a lack of CTR of Hfq resulted in lower resistance than the absence of the whole *hfq* gene. These results might suggest that Hfq is involved in negative regulation of expression of the *kan* gene. In fact, under steady-state conditions, the level of *kan* mRNA was significantly (about four times) higher in the Δ*hfq*::*kan* mutant than in the *hfq*^+^ strain (Figure 10). This effect was also observed in the ΔCTR*hfq*::*kan* mutant, though it was less pronounced (about 2-fold higher level relative to *hfq*^+^). Since Hfq is an RNA chaperone acting mostly on small RNA regulatory species [2,3,4], one could suggest that post-transcriptional events are involved in this regulation. However, the degradation rate of *kan* mRNA was similar in the Δ*hfq*::*kan* and *hfq*^+^ strains, while it was more rapid in the ΔCTR*hfq*::*kan* mutant. This indicated that differences in mRNA stabilities are not the sole cause of differences in the resistance to high kanamycin concentration. Thus, regulation of the *kan* gene expression by Hfq must involve also other stages, apart from RNA decay. On the other hand, decreased kanamycin resistance of the mutant devoid of CTR may indicate involvement of changes in DNA topology in such a regulatory process, since this region of Hfq has been demonstrated to interact with DNA and to change its conformation, including compaction and condensation [5,9].

Opposite results were obtained in experiments with p27cmr plasmid-borne resistance to a high concentration (204 μg/mL) of chloramphenicol. In this case, defects in the *hfq* gene resulted in impairment of antibiotic resistance relative to the wild-type bacteria (Table 4). The down-regulation of expression of chloramphenicol resistance was more pronounced in the Δ*hfq*::*kan* mutant than in the ΔCTR*hfq*::*kan* strain, suggesting that the entire Hfq protein may be involved in this regulation, and partial remaining of its function in the latter mutant might allow to enhance the expression of the chloramphenicol resistance to some extent. These effects might be caused by impaired replication of lambdoid plasmid p27cmr in the presence of the Δ*hfq*::*kan* mutation, and intermediate effect of the ΔCTR*hfq*::*kan* mutation, as concluded from significantly lower efficiency of transformation of the mutant strains in comparison to the *hfq*^+^ bacteria (Table 3).

When pSC101 (a low copy number plasmid)-borne tetracycline resistance was investigated, some unexpected results were obtained. A concentration of 68 μg/mL of tetracycline caused high sensitivity (no bacterial colonies could be observed) of the Δ*hfq*::*kan* host, while both *hfq*^+^ and ΔCTR*hfq*::*kan* expressed relatively high resistance (Table 5). This suggests that NTR of Hfq may be responsible for positive regulation of expression of the *tet* gene. When pSC101 was present in cells, steady-state levels of *tet* mRNA were similar in Δ*hfq*::*kan* and *hfq*^+^ bacteria, and decreased in the ΔCTR*hfq*::*kan* mutant, while stability of this mRNA was increased in Δ*hfq*::*kan* cells relative to both *hfq*^+^ and ΔCTR*hfq*::*kan* strains (Figure 10). These results indicate that effects of Hfq on *tet* mRNA decay cannot explain the different phenotypes of tetracycline resistance. Interestingly, when *tet* was present on a medium copy number plasmid pBR322, resistance to tetracycline (at 68 μg/mL) of all tested strains (*hfq*^+^, Δ*hfq*::*kan* and ΔCTR*hfq*::*kan*) was comparable (Table 6). Therefore, we suggest that higher copy number of *tet* might compensate for the decreased level of its expression under conditions of dysfunction of CTR. On the other hand, the same copy number of pBR322 could not prevent impaired resistance to ampicillin (at 5 mg/mL) of the Δ*hfq*::*kan* mutant (Table 7). Definitely, different mechanism of the control of gene expression by Hfq operate for *tet* and *bla*.

Since the involvement of Hfq in the regulation of ColE1-like plasmid DNA replication was suggested [11], one might ask whether observed differences in antibiotic resistance could arise from various plasmid copy numbers in wild-type cells and *hfq* mutants. However, real time quantitative PCR analysis indicated a very similar number of pBR322 molecules per cell in all investigated host strains (Table 8). The highest plasmid copy number was detected at late exponential/early stationary phase of bacterial growth, which corresponds to previously reported elevated levels of the Hfq protein in the stationary phase of bacterial cultivation [11]. However, pBR322 copy number was independent of the *hfq* gene function (Table 8), indicating that Hfq is not crucial in this aspect of ColE1-like plasmid biology. On the other hand, efficiency of transformation of host cells, which can be considered as an indirect and rough measure of plasmid replication, with pBR322 was decreased in *hfq* mutants relative to *hfq*^+^ bacteria (Table 3). Therefore, we suggest that Hfq may be involved in establishment of ColE1-like plasmid replication after its entrance into the host cell. Interestingly, despite a lack of significant differences between efficiency of transformation of Δ*hfq*::*kan* and ΔCTR*hfq*::*kan* cells with pBR322, the presence of such differences was evident when a derivative of this plasmid, devoid of the *rom* gene coding for a negative regulator of ColE1-like plasmid replication initiation, acting by stabilizing RNA-RNA interactions, was used (Table 3). These results suggest that there may be an interplay between Hfq and Rom at the stage of pBR322 replication initiation process. In fact, both Hfq [2,3,4] and Rom [32,33] modulate RNA-RNA interactions, and such interactions are crucial in the control of ColE1-like plasmid DNA replication [32,33]. In addition, DNA-binding activity of Hfq and its ability to change DNA topology [12,13,14,15] might also considerably influence the initial steps of replication of such plasmids.

To assess whether resistance of tested strains to high antibiotic concentrations results from recovering of the cell population after initial killing of a large fraction bacteria or the starting population is already resistant and not affected, we have tested the growth of strains in liquid media supplemented with tested antibiotics. This allowed us to address an important question of the character of bacterial response to such conditions, as summarized previously [31]. Our results demonstrated that in the case of chromosome-borne kanamycin resistance (at 233 μg/mL) and the plasmid (p27cmr)-borne resistance to chloramphenicol (at 204 μg/mL), the insensitivity of cells to high antibiotic levels is an intrinsic feature of bacteria rather than a kind of developing antibiotic tolerance (Figure 3 and Figure 4). A similar phenomenon was observed when testing resistance of *hfq*^+^/pSC101 to 68 μg/mL tetracycline (Figure 5). However, the ΔCTR*hfq*::*kan*/pSC101 mutant required a long adaptation time to restore the initially inhibited growth after supplementation of the medium with 68 μg/mL tetracycline (Figure 5). The Δ*hfq*::*kan*/pSC101 mutant did not form colonies on plates with this concentration of the tested antibiotic, however, following initial growth inhibition of the culture, restoration of bacterial propagation could be observed (Figure 5). These results were corroborated by measurement of minimal inhibitory concentrations (MIC) of tetracycline in liquid media, which were high (1024 μg/mL or higher) for all tested strains (Figure 5). One can suggest that either this arose from isolation of a suppressor mutant or growth conditions in solid and liquid media differ enough from each other to block bacterial replication in the former medium and to support it in the latter one, in the presence of a high level of tetracycline. Quite similar kinetics of bacterial culture growth in the liquid medium containing 68 μg/mL tetracycline were observed for Δ*hfq*::*kan* and ΔCTR*hfq*::*kan* mutants (but not the *hfq*^+^ host) bearing the pBR322 plasmid, revealing higher copy number than pSC101 (Figure 6, Table 1). This suggests that high level tetracycline resistance develops with the specific mechanism requiring the adaptation period. Interestingly, the delay in growth of these mutants under described conditions was alleviated when a pBR322 derivative devoid of the functional *rom* gene was used (Figure 7). Therefore, these results corroborate the proposal, presented in the preceding paragraph, that there is a functional interplay between Hfq and Rom proteins. Liquid medium experiments performed with the medium containing 5 mg/mL ampicillin indicated immediate growth of each tested bacterial strain harboring either pBR322 or its *rom*-devoid derivative (Figure 8 and Figure 9). This confirms that tetracycline resistance conditions are specific and that they differ from those caused by other antibiotics.

Since kanamycin-resistance (*kan*) and tetracycline-resistance (*tet*) genes, investigated in this study, derive from Tn*5* and Tn*10*, respectively, it is worth mentioning that Hfq has been demonstrated to regulate the efficiency of transposition of these mobile genetic elements. A considerable suppression of Tn*5* transposition by the Hfq protein was demonstrated, and this negative control was shown to be due to inhibition of expression of the gene coding for IS50 transposase [34]. This inhibition is, however, indirect as it is mediated by the regulation of Crp (cyclic AMP receptor protein) [34]. Transposition of Tn*10* has also been demonstrated to be down-regulated by Hfq, while this inhibition is mediated by interaction between the *hfq* gene product and the IS10 transposase-encoding mRNA (particularly the ribosome-binding site), which results in translation repression [35]. Interestingly, ChiX, a small RNA molecule, was shown to titrate Hfq out, facilitating translation of the IS10 transposase mRNA [35]. Although these reports clearly indicate that Hfq may influence spreading of Tn*5* and Tn*10*, it seems unlikely that this mechanism might contribute to differential antibiotic-resistance of *hfq* mutants, observed in this study, since the transposition process is generally rare, even if stimulated in the absence of the Hfq functions.

The major unanswered question remains how can Hfq regulate the expression of bacterial resistance to high antibiotic concentrations. Definitely, observed differences in stabilities of mRNAs derived from genes coding for proteins responsible for resistance to tetracycline and kanamycin between *hfq*^+^ bacteria and *hfq* mutants (Figure 10) are too low to explain this phenomenon. Therefore, it is likely that the Hfq protein, and also its CTR and/or NTR parts, may be involved in the regulation of gene expression at other levels. This resembles results published earlier by Le Derout et al. [36], who demonstrated that significant differences in levels of transcripts derived from *rpsO*, *rpsT*, and *rpsB-tsf* genes, between *hfq*^+^ and *hfq* mutant bacteria, did not result from various posttranscriptional modifications, like mRNA degradation. Interestingly, levels of some other RNA species, like *lpp* and *pnp* mRNAs or tRNA transcripts, were unaffected by the absence of Hfq [36]. Moreover, translation regulation of *rpsO*, *rpsT,* and *rpsB-tsf* was also not changed in the tested strains [36]. Therefore, RNA decay-independent regulation of gene expression by Hfq appears specific to some transcripts, which is fully compatible with the results presented in this report. Although mechanism(s) of this Hfq-mediated regulation remain(s) to be elucidated, our results corroborate the previously reported proposals that the *hfq* gene product is involved in more controlling processes than only those related to its RNA chaperone functions [4,5,9,10,12,13,14,36]. These include various stages of gene expression.

One should also take into consideration specific stress conditions that are likely caused by the use of high concentrations of antibiotics. Since Hfq is involved in different stress responses [2,3,4,5], especially those involving small RNA and mRNA interactions, one might speculate that differences in antibiotic resistance events, observed between *hfq*^+^ bacteria and *hfq* mutants, might have arose indirectly from intensive Hfq-mediated reactions (or their absence in the mutants) which proceeded in response to the stress conditions. Thus, Hfq might participate mainly in such reactions that could influence its normal functions in the regulation of gene expression processes.

In this light, it is interesting that the growth inhibition of *hfq*^+^-*kan* and ΔCTR*hfq*::*kan* strains in the liquid medium supplemented with high kanamycin concentration was more pronounced at the stationary phase than at the exponential phase (Figure 3). This could suggest an involvement of the stationary phase stress conditions. In fact, an interplay between functions of *rpoS* (coding for the stationary phase σ factor) and *hfq* were reported. Especially, *rpoS* expression is regulated by several small RNA molecules that interact with mRNA derived from this gene, and in the absence of Hfq activity, *rpoS*-specific mRNA levels are reduced and efficiency of its translation is affected [37,38,39,40,41]. Moreover, RpoS and Hfq may participate together in the cellular response to stress conditions [42]. Therefore, influence of the deficiency in RpoS, and the resultant disturbed stress response, on resistance to high concentrations of antibiotics in the absence of Hfq or its CTR part appears likely.

One might also speculate that changes in cellular stress responses could participate in the delay of growth of Δ*hfq*::*kan* and ΔCTR*hfq*::*kan* mutants bearing pSC101, pBR322 or pBR322Δ*rom* in liquid media, after transferring the cultures to broth containing high tetracycline concentrations (Figure 5, Figure 6 and Figure 7). Since Hfq is required for efficient general stress response through interactions with various regulatory RNAs, and it indirectly stimulates the production of RpoS, the major player in this response [2,3,4,5,37,38,39,40,41], perturbations caused by poorly managed stress, which are caused by a high tetracycline level, might eighter significantly slow the growth of *hfq* mutants down or delay their adaptation to these growth conditions.

It is also striking that effects of deletions of the whole *hfq* gene and part of this gene coding for CTR were different in almost all experiments performed under conditions of high concentrations of antibiotics. This might suggest that CTR and/or NTR alone could negatively or positively regulate resistance to antibiotics (depending on the kind of antimicrobial agent). Such a hypothesis signals a possibility of independent biological activities of both regions of the Hfq protein.

## 4. Materials and Methods

### 4.1. Bacterial Strains

*E. coli* strains derived from MG1655 [43] were used in this work. The strains bearing a wild-type *hfq* gene linked to the *kan* marker (called *hfq*^+^ in this report), and Δ*hfq*::*kan* and ΔCTR*hfq*::*kan* mutants, were constructed using the λ-Red system coupled with restriction-independent PCR mutagenesis, as described previously [44]. The *hfq* gene region was amplified with PCR, using genomic DNA of *E. coli* MG1655 and primers 1 and 2 (Table 9). The reaction product was used as a megaprimer in a subsequent PCR, to introduce *hfq* into plasmid pKD13 (used as a template) [45], downstream of the FRT site. The reaction product was digested with DpnI, and used for transformation of *E. coli* BW25141 strain [45]. The pKD13*hfq* plasmid, with deletion of the *hfq* gene fragment coding for CTR, was constructed by using PCR with primers 3 and 4 (Table 9) to produce a megaprimer. Then, a new construct was obtained as described above, but pKD13*hfq* was used as a template. The recombinant plasmids were used to obtain linear DNA molecules for transformation of electrocompetent *E. coli* MG1655 cells bearing the pKD46 plasmid with the λ-Red system [45]. The plasmid was removed by incubation of bacteria at 37 °C. To synthesize *hfq*::*kan* and ΔCTR::*kan* inserts, PCR reactions with primers 5 and 6 (Table 9) were performed. Analogous reactions with primers 6 and 7 (Table 9) were conducted to obtain the *kan* marker located near the wild-type *hfq* gene. Following transformation, obtained clones were verified for the presence of *hfq*^+^, Δ*hfq*::*kan* or ΔCTR*hfq*::*kan* alleles by DNA sequencing.

### 4.2. Plasmids

Plasmids p27cmr [46], pSC101 [47], and pBR322 [48] were used. To construct plasmid pBR322Δ*rom*, PCR reaction with primers 8 and 9 (Table 8) and pBR322 DNA as a template were performed, to obtain a whole length original plasmid devoid of the *rom* gene. The *cat* gene was amplified by PCR using pBAD33 [49] as a template and primers 10 and 11 (Table 9). Following DNA ligation and transformation of *E. coli* cells, selected clones were used to verify the construction by DNA sequencing.

### 4.3. Media and Growth Conditions

Bacteria were cultured in LB medium [50] at 37 °C with agitation. Growth of bacterial culture was monitored by measurement of OD_600_. Plates contained LB medium supplemented with 1.5% bacteriological agar. Media were supplemented with antibiotics at the indicated concentrations.

### 4.4. Antibiotic Resistance Estimated on Plates with Solid Medium

Bacterial strains were cultured in LB medium at 37 °C. At mid-log phase, samples of cultures were withdrawn and titrated on LB plates and LB plates containing the indicated antibiotic at the indicated concentration. The fraction of antibiotic-resistant cells was calculated by dividing the titer of bacteria on LB with antibiotic by titer of bacteria on LB without antibiotic (which corresponded to 5 × 10^8^ colony forming units (CFU) per mL, and this value was the same in plates with standard antibiotic concentrations) and multiplied by 100%. Experiments were repeated six times, and the results are presented as mean values ± SD.

### 4.5. Monitoring of Growth in Liquid Medium

Bacteria were grown in LB medium at 37 °C, and at mid-log phase, the cultures were diluted 10 times in either LB or LB with a high concentration of one of the tested antibiotics (233 μg/mL kanamycin, 204 μg/mL chloramphenicol, 68 μg/mL tetracycline or 5 mg/mL ampicillin). Culture growth was monitored by measurement of OD_600_ at the indicated time intervals. 

### 4.6. Efficiency of Transformation

Transformation efficiency of bacterial cells with plasmid DNA was tested using the calcium chloride method [50]. Competent *E. coli* cells (0.1 mL suspension in 50 mM CaCl_2_) were incubated in ice with plasmid DNA for 60 min, transferred to 43 °C for 3 min, and cultured with agitation for 60 min at 37 °C. For titration, serial dilutions in 0.9% NaCl were spread onto LB plates with or without antibiotic at standard concentration (34 μg/mL chloramphenicol, 12.5 μg/mL tetracycline, 50 μg/mL kanamycin, 50 μg/mL ampicillin). Plates were incubated overnight at 37 °C. Efficiency of transformation was calculated as number of transformants per 1 μg of plasmid DNA.

### 4.7. Plasmid Copy Number

Copy number of plasmid DNA in bacterial cells was determined by real time quantitative PCR, as described previously [51]. Briefly, samples of bacterial cultures (1.5 mL) were withdrawn, centrifuged in a microfuge, and pellets were washed and dissolved in 0.5 mL of water. Cells were lysed by boiling for 5 min. The quantitative PCR reactions were conducted using primers 12 and 13 (Table 7) for chromosomal *mreB* gene, and primers 14 and 15 (Table 8) for plasmid *tet* gene. Analysis of data was conducted using the E-Method [51,52]. The results are expressed as number of copies of plasmid-located gene (*tet*) per number of copies of chromosomally-located gene (*mreB*), assumed to be equal to copies of plasmid DNA per chromosomal DNA. Experiments performed using LB medium without antibiotic and with ampicillin (50 μg/mL) gave similar results (with no statistically significant differences).

### 4.8. Northern-Blotting

Bacteria were cultured in LB medium with the appropriate antibiotic at 37 °C with agitation. The overnight culture was diluted 1:100 in a fresh LB medium and cells were grown until the OD_600_ of 0.5. Samples were withdrawn at time “0”, and rifampicin was added to a final concentration of 200 µg/mL. To each sample (10^8^ cells; after 2, 4, 8, 16, 32 min), ice-cold 96% ethanol/5% phenol (1:1 *v*/*v*) mixture was added immediately to prevent mRNA degradation. The cells were then harvested by centrifugation, and the pellets were frozen in liquid nitrogen. RNA was extracted with TRIzol^®^ Max™ Bacterial RNA Isolation Kit (Thermo Scientific, Waltham, MA, USA) according to the manufacturer’s instruction. RNA was quantified using a NanoDrop Spectrophotometer (Thermo Scientific, USA) and visually assessed after electrophoretical separation in 1% agarose gel. RNA samples were mixed with 2 × loading buffer (5 mM EDTA, 0.1% bromophenol blue, 0.1% xylene cyanol, and 95% formamide), and heated at 70 °C for 5 min. 1% agarose gels, stained with 0.5 μg/mL ethidium bromide, were run at 50 V for approximately 1 h in TBE buffer (Thermo Scientific, USA). The RNAs were then transferred onto BrightStarTM+ (positively charged nylon) membranes (Invitrogen, Waltham, MA, USA) using a Bio-Rad transblot apparatus (Bio-Rad, Hercules, California, USA). Next, the membranes were crosslinked at 1.2 mJ for 5 min and dried at 50 °C for 30 min to improve sensitivity. Before hybridization, the membranes were pre-hybridized for 30 min at 42 °C in ULTRAhybTM—Oligo Hybridisation Buffer (Invitrogen, USA). Then, the pre-hybridization buffer was removed, and buffer containing 50 pmol/mL labeled probe (probe TetpSC101 (5′-Biotin-CGAGCCCGATCTTCCCCATCGGTGATGTCG) for tet mRNA, and probe KanpKD13 (5′-Biotin-GGAACGCCCGTCGTGGCCAGCCACGATAGC) for kan mRNA) was added. The probes were modified with biotin at the 5′ terminus, and were synthesized and purified via HPLC by the Genomed Company (Warsaw, Poland). The membranes were hybridized overnight at 42 °C with gentle shaking and subsequently rinsed with washing buffer (1× SSC (Sigma Aldrich, Burlington, MA, USA), 0.1% SDS) three times for 10 min at room temperature. Following hybridization, membranes were blocked again with ULTRAhybTM—Oligo Hybridisation Buffer (Invitrogen, Waltham, MA, USA) for 15 min with gentle shaking at room temperature, followed by incubation for an additional 30 min with the hybridization buffer containing stabilized streptavidin-HRP conjugate (Merc Millipore, Burlington, MA, USA). After washing four times (1× SSC, 0.1% SDS), the membranes were incubated in PierceTMECL Western Blotting Substrate (Thermo Scientific, Waltham, MA, USA) for 5 min. Finally, the membranes were placed in a cassette with X-ray films (Merc Millipore, Burlington, MA, USA) and exposed for different times (depending on the desired signal intensity). The intensities of bands were analyzed with the QuantityOne software (Bio-Rad, Hercules, CA, USA). The results were calculated as a ratio of amount of tested mRNA to the amount of rRNA (assessed by densitometry of bands visualized in agarose gels with ethidium bromide).

### 4.9. Determination of Minimal Inhibitory Concentration (MIC) of Antibiotics

To determine minimal inhibitory concentration (MIC) of all tested antibiotics (kanamycin, chloramphenicol, tetracycline, and ampicillin), overnight cultures of tested bacteria were diluted 1:100 in fresh LB medium containing increasing concentrations of investigated antibiotic, and incubated in 96-well plates for 16 h at 37 °C. The MIC value was considered as the lowest antibiotic concentration causing complete inhibition of bacterial growth.

### 4.10. Determination of Levels of Hfq Proteins

Levels of the Hfq protein and its NTR were determined in the *hfq*^+^ strain and the ΔCTR*hfq*::*kan* mutant, respectively (no Hfq protein could be detected in the Δ*hfq*::*kan* mutant). Amounts of Hfq or NTR were estimated using standard Western-blotting procedure, as described previously [50]. Specific anti-Hfq antibody (against Hfq from *E. coli* K-12) was used (cat. no. X-P0A6 × 3-N; Abmart Yiyao Keyi Co. Ltd., Shanghai, China).

### 4.11. Statistical Analysis

Statistical significance of differences in mean values of two compared experimental systems was assessed using the *t*-test. Statistically significant differences were assessed to occur when *p* < 0.05.

## 5. Conclusions

Differential chromosome-borne and plasmid-borne resistance to various antibiotics, at concentrations significantly higher than those used in standard laboratory practice, was found in *E. coli* strains bearing wild-type *hfq* allele and mutations in the *hfq* gene. Genetic analysis suggested that CTR and/or NTR of Hfq may be involved in regulation of expression of different antibiotic resistance genes independently. Such a regulation appears to be independent of the control of mRNA decay. Moreover, Hfq may participate in the control of ColE1-like plasmid DNA replication, especially at the initiation stage (possibly interplaying with the plasmid-encoded Rom protein), and during plasmid establishment after transformation of bacterial cells. 

## Figures and Tables

**Table 1:** Plasmids used in this work.

Plasmid	Replicon	Marker(s)	Copy Number/Cell
p27cmr	Lambdoid	*cat* (Cm^R^)	Low (6–7)
pSC101	pSC101	*tet* (Tet^R^)	Low (~5)
pBR322	pMB1 (ColE1-like)	*bla* (Ap^R^), *tet* (Tet^R^)	Medium (20–40)
pBR332Δ*rom*	pMB1 (ColE1-like)	*bla* (Ap^R^), *tet* (Tet^R^), *cat* (Cm^R^)	High (~100)

**Table 2:** Resistance of *E. coli hfq*^+^, Δ*hfq*::*kan,* and ΔCTR*hfq*::*kan* strains to different concentrations of kanamycin.

Strain	Percent of Resistant Cells ^1^	
	50 μg/mL Kanamycin	233 μg/mL Kanamycin
*hfq* ^+^	100%	0.012 ± 0.003%
Δ*hfq*::*kan*	100%	49.64 ± 5.11%
ΔCTR*hfq*::*kan*	100%	1.26 ± 0.29%

^1^ These values were calculated as the titer of the culture (in colony forming units per ml) determined on plates with kanamycin divided by titer on plates with no antibiotic × 100%. When the titer of the bacterial culture was undistinguishable on plates with and without antibiotic, the value was considered as 100%. The presented values are mean values from six experiments ± SD. Statistically significant differences (*p* < 0.05) were found for all pairs of mean values at 233 μg/mL kanamycin.

**Table 3:** Efficiency of transformation of *E. coli hfq*^+^, Δ*hfq*::*kan* and ΔCTR*hfq*::*kan* strains with plasmids p27cmr, pSC101, pBR322, and pBR322Δ*rom*.

Strain	Efficiency of Transformation (Transformants/μg DNA) ^1^			
	p27cmr	pSC101	pBR322	pBR322Δ*rom*
*hfq* ^+^	1.09 ± 0.42 × 10^5^	1.72 ± 0.13 × 10^4^	8.57 ± 0.61 × 10^4^	2.58 ± 0.34 × 10^5^
Δ*hfq*::*kan*	1.21 ± 0.19 × 10^3^ (*)	2.03 ± 0.48 × 10^3^ (*)	1.53 ± 0.22 × 10^4^ (*)	5.60 ± 0.18 × 10^3^ (*)
ΔCTR*hfq*::*kan*	1.39 ± 0.25 × 10^4^ (* #)	3.11 ± 0.37 × 10^3^ (*)	3.04 ± 0.16 × 10^4^ (*)	6.51 ± 0.75 × 10^4^ (* #)

^1^ Values were calculated as number of transformants per 1 μg of plasmid DNA, when employing standard concentrations of antibiotics (34 μg/mL chloramphenicol, 12.5 μg/mL tetracycline, and 50 μg/mL ampicillin) in the plates with selective medium. The presented values are mean values from three experiments ± SD. Statistically significant differences (*p* < 0.05) between results obtained for *hfq*^+^ vs. *hfq* mutant hosts (*) and for Δ*hfq*::*kan* vs. ΔCTR*hfq*::*kan* host (#) are indicated.

**Table 4:** Resistance of *E. coli hfq*^+^, Δ*hfq*::*kan,* and ΔCTR*hfq*::*kan* strains bearing the p27cmr plasmid to different concentrations of chloramphenicol.

Strain	Percent of Resistant Cells ^1^	
	34 μg/mL Chloramphenicol	204 μg/mL Chloramphenicol
*hfq*^+^/p27cmr	100%	88.00 ± 5.66%
Δ*hfq*::*kan*/p27cmr	100%	0.06 ± 0.02%
ΔCTR*hfq*::*kan*/p27cmr	100%	0.48 ± 0.17%

^1^ Values were calculated as the titer of the culture (in colony forming units per ml) determined on plates with chloramphenicol divided by the titer on plates with no antibiotic × 100%. When the titer of the bacterial culture was undistinguishable on plates with and without antibiotic, the value was considered as 100%. The presented values are mean values from six experiments ± SD. Statistically significant differences (*p* < 0.05) were found for all pairs of mean values at 204 μg/mL chloramphenicol.

**Table 5:** Resistance of *E. coli hfq*+, Δ*hfq*::*kan,* and ΔCTR*hfq*::*kan* strains bearing the pSC101 plasmid to different concentrations of tetracycline.

Strain	Percent of Resistant Cells ^1^	
	12.5 μg/mL Tetracycline	68 μg/mL Tetracycline
*hfq*^+^/pSC101	100%	17.69 ± 3.27%
Δ*hfq*::*kan*/pSC101	100%	<0.0001%
ΔCTR*hfq*::*kan*/pSC101	100%	38.88 ± 5.19%

^1^ These values were calculated as the titer of the culture (in colony forming units per ml) determined on plates with tetracycline divided by titer on plates with no antibiotic × 100%. When the titer of the bacterial culture was undistinguishable on plates with and without antibiotic, the value was considered as 100%. The presented values are mean values from six experiments ± SD. Statistically significant differences (*p* < 0.05) were found for all pairs of mean values at 68 μg/mL tetracycline.

**Table 6:** Resistance of *E. coli hfq*+, Δ*hfq*::*kan*, and ΔCTR*hfq*::*kan* strains bearing the pBR322 plasmid to different concentrations of tetracycline.

Scheme 1	Percent of Resistant Cells ^1^	
	12.5 μg/mL Tetracycline	68 μg/mL Tetracycline
*hfq*^+^/pBR322	100%	86.06 ± 10.44%
Δ*hfq*::*kan*/pBR322	100%	71.09 ± 12.03%
ΔCTR*hfq*::*kan*/pBR322	100%	78.59 ± 16.66%

^1^ These values were calculated as the titer of the culture (in colony forming units per ml) determined on plates with tetracycline divided by titer on pates with no antibiotic × 100%. When the titer of the bacterial culture was undistinguishable on plates with and without antibiotic, the value was considered as 100%. The presented values are mean values from six experiments ± SD. No statistically significant differences (*p* > 0.05) were found for any pairs of mean values at 68 μg/mL tetracycline.

**Table 7:** Resistance of *E. coli hfq*+, Δ*hfq*::*kan,* and ΔCTR*hfq*::*kan* strains bearing the pBR322 plasmid to different concentrations of ampicillin.

Strain	Percent of Resistant Cells ^1^	
	50 μg/mL Ampicillin	5 mg/mL Ampicillin
*hfq*^+^/pBR322	100%	69.76 ± 8.07%
Δ*hfq*::*kan*/pBR322	100%	9.00 ± 1.82%
ΔCTR*hfq*::*kan*/pBR322	100%	87.90 ± 10.29%

^1^ These values were calculated as the titer of the culture (in colony forming units per ml) determined on plates with ampicillin divided by titer on plates with no antibiotic × 100%. When the titer of the bacterial culture was undistinguishable on plates with and without antibiotic, the value was considered as 100%. The presented values are ean values from six experiments ± SD. Statistically significant differences (*p* < 0.05) were found for *hfq*^+^ vs. Δ*hfq*::*kan*, for Δ*hfq*::*kan* vs. ΔCTR*hfq*::*kan*, and for *hfq*^+^ vs. ΔCTR*hfq*::*kan*.

**Table 8:** Plasmid pBR322 copy number in *E. coli hfq*^+^, Δ*hfq*::*kan,* and ΔCTR*hfq*::*kan* strains at different phases of bacterial culture growth.

Strain	OD_600_ of Bacterial Culture	pBR322 Copy Number ^1^
*hfq*^+^/pBR322	0.3	18.60 ± 1.84
	0.8	37.98 ± 1.11
	1.4	21.31 ± 1.76
Δ*hfq*::*kan*/pBR322	0.3	23.30 ± 1.02
	0.8	41.09 ± 2.37
	1.4	27.80 ± 1.90
ΔCTR*hfq*::*kan*/pBR322	0.3	21.77 ± 2.16
	0.8	43.07 ± 2.94
	1.4	27.03 ± 4.08

^1^ These values were calculated as a ratio between number of plasmid DNA and chromosomal DNA molecules, calculated on the basis of qPCR reactions with primers specific to plasmid and chromosomal genes. The presented values are mean values from six experiments ± SD. Statistically significant differences (*p* < 0.05) were found for results obtained for OD_600_ 0.3 vs. 0.8, and 0.8 vs. 1.4 in each host strain, but not for any other values. Experiments were performed using LB medium without antibiotic and with ampicillin (50 μg/mL); no statistically significant differences were detected between results obtained under these two experimental conditions.

**Table 9:** Primers used in this work.

Primer Name	Sequence	Description
Primer 1	5′-CGG GGA TCC GTC GAC CTG CAG TAT GGC TAA GGG GCA ATC TTT AC	Forward primer containing a sequence complementary to the 5′-region of *hfq* and a specific sequence of pKD13
Primer 2	5′-AAG TTC CTA TAC TTT CTA GAG AAT AGG AAC TTC GAT TAT TCG GTT TCT TCG CTG TCC	Reverse primer containing a sequence complementary to the 5′-region of *hfq* and a specific sequence of pKD13
Primer 3	5′-ACG CGA TTT CTA CTG TTG TCC CGT AAT CGA AGT TCC TAT TCT CTA GA	Forward primer containing a sequence complementary to the distal part of NTR of *hfq* on pKD13 and the termination sequence
Primer 4	5′-TCA AAA GCG CTC TGA AGT TCC TAT ACT TTC TAG AGA ATA GGA ACT TCG ATT ACG	Reverse primer containing a sequence complementary to the distal part of NTR of *hfq* on pKD13 and the termination sequence
Primer 5	5′-TCA GAA TCG AAA GGT TCA AAG TAC AAA TAA GCA TAT AAG GAA AAG AGA GAA TGG CTA AGG GGC AAT CTT T	Forward primer containing a sequence complementary to the5′-region of *hfq* and overhang complementary to the *hfq* region on *E. coli* MG1655 genome
Primer 6	5′-CGG GGA ACG CAG GAT CGC TGG CTC CCC GTG TAA AAA AAC AGC CCG AAA CCT GTG TAG GCT GGA GCT GCT TC	Reverse primer containing a sequence complementary to the 5′-region of *hfq* and overhang complementary to the *hfq* region on *E. coli* MG1655 genome
Primer 7	5′-TCA GAA TCG AAA GGT TCA AAG TAC AAA TAA GCA TAT AAG GAA AAG AGA GAA TGA TTG AAC AAG ATG GAT TG	Forward primer containing a sequence complementary to the 5′-region of *kan* and an overhang complementary to the *hfq* region on *E. coli* MG1655 genome
Primer 8	5′-CAC ATG CAG CTC CCG GAG ACG	Forward primer for amplification of pBR322 without *rom*
Primer 9	5′-TGA TGC CTC CGT GTA AGG GGG	Reverse primer for amplification of pBR322 without *rom*
Primer 10	5′-TTA AAA AAA TTA CGC CCC GCC	Forward primer for amplification of the *cat* gene
Primer 11	5′-ATG GAG AAA AAA ATC TCT GGA	Reverse primer for amplification of the *cat* gene
Primer 12	5′-ACC GCA GAA CGT ATC AAG CA	Forward primer for amplification of the *mreB* gene
Primer 13	5′-GG TAA AAC CGC GTG GAA CAC	Reverse primer for amplification of the *mreB* gene
Primer 14	5′-CTC ATC GTC ATC CTC GGC AC	Forward primer for amplification of the *tet* gene
Primer 15	5′-TAG CAG CAC GCC ATA GTG AC	Forward primer for amplification of the *tet* gene

## Data Availability

Raw data are available from authors on request.
